# Improving the job market in economics (and beyond…)

**DOI:** 10.1073/pnas.2609971123

**Published:** 2026-05-04

**Authors:** Peter L. Rousseau

**Affiliations:** ^a^ Department of Economics, Vanderbilt University, Nashville, TN 37235

The convening of an annual job market for fresh or soon-to-be-minted Ph.D. economists at the meeting of the Allied Social Science Associations (ASSA), the world’s largest gathering of professional economists that had grown to more than 12,000 participants by 2020, can be viewed as a triumph in organic market design. Starting in the early 1970s, in-person first-round interviews for entry-level faculty positions in the United States and many other countries were almost universally conducted there. In that mechanism, hiring committees of academic departments (and research institutions) would read literally hundreds of application packets from job candidates, with nearly all arriving in late November or early December, and then schedule 20 to 30 interviews at the ASSA convention in early January from among the applicants (see ref. 1 for additional details). As any scholar or practitioner of financial markets and their history understands, the concentration of such a market at a single moment (and in this case location) can offer a leap in liquidity, and in this case led to reasonably efficient employer–candidate matches and outcomes given that the first-round interviews acted as a screening device for later campus visits and job offers. The article by Qiu et al. (2) reports on an intervention with the potential to improve upon these matches.

In contrast to the usual focus on the advantages of a call market’s liquidity in thin markets, however, it is interesting to note that the job market in economics is quite thick, and that this thickness can lead to congestion. By congestion, I mean problems related to the constraints imposed by a three-day convention and the number of candidates a department can interview there, along with the associated possibility of departments coming up empty at the conclusion of a recruiting season due to their inability to identify close and willing matches. To lessen the congestion, some sharing of information about candidates and potential matches would make its way around the convention through social gatherings, elevator conversations, and other means as the first round of interviews played out. The reputations of the economists sharing such information had some bearing on how certain candidates were perceived by those recruiters receiving it and could be viewed as a supplement to the actual reference letters prepared by candidates’ dissertation advisors and committee members.

The mechanism described above does not warrant excessive self-congratulation, however, due to elements of a “star” factor that have always seemed clear to those viewing the process. With an emphasis on leading candidates from the very top institutions, some departments would overreach in attempting to secure such candidates, and by March would find themselves without a viable match. Candidates from institutions outside of the very top, and those with less well-known mentors, could struggle to gain visibility. Given that many very fine candidates do not hail from the highest-ranked departments, the de-emphasis of such candidates in the matching process, combined with a reduced access to the informal information network that many recruiters from lower-ranked departments faced, could lead to the inefficient outcome of jobs ultimately being left on the table. With so little time between the arrival of application packets and decisions about which candidates to interview at the convention, it was often practical to rely upon letters from known advisors in this first stage, leaving some candidates at a disadvantage, especially in the absence of systematic ways to promote them.

The onset of the COVID-19 pandemic in March 2020 led the ASSA convention to move online in 2021 and 2022, with first-round interviews conducted remotely. When it became clear that remote interviews were at least nearly as effective as in-person ones and did not require costly travel in early January for all job candidates and interviewers, the American Economic Association (AEA), which organizes the ASSA meetings, for this and a number of other reasons, recommended that departments no longer conduct first-round interviews at the convention. This meant that much of the informal information shared about job candidates there would no longer be so readily available to well-connected recruiters.

In this context, the authors make a welcome and useful contribution to the market design literature with a fascinating experiment designed to substitute for and even improve upon the informal information channels lost to the economics job market in the new postpandemic normal. Given that some job candidates are less active self-promoters than others and that, conversely, excessive self-promotion can in some cases be viewed as a negative by prospective recruiters, the authors’ proposed mechanism offers serious promise for leveling the playing field, even if just modestly, for economics job candidates in terms of their visibilities, and perhaps even for expanding the number of jobs actually filled over the course of a recruiting season.

In the experiment, an AI-based algorithm, supplemented with some human checking and reassignments, matched selected economists on social media (i.e., the “influencers”) with willing job candidates based on the closeness of their research. About 43 percent of willing candidates were selected for this treatment. The key to the experiment lies in the matches themselves, which were assigned in a manner that did not take the relative prominence or institutional ranking of an influencer directly into account. All candidate participants were invited to post a tweet about their job market papers on a social media site created for this purpose, and the influencers were asked to post neutral quote-tweets about the members of the treatment group to which they had been assigned. If executed according to design, recruiters viewing the quote-tweets receive information about the closeness of a given candidate’s research interests to those of the influencer. This may function as a partial substitute for the painstaking process of deducing such information across the hundreds of application packets that recruiters receive with only a brief period for making initial decisions. Knowing that a candidate’s research is close to that of Professor “X” is a tangible signal that could make that candidate more likely to be interviewed or receive a campus flyout or job offer from an institution seeking an entry-level economist like Professor X. The experiment indicates that individuals in the treatment group did indeed receive more campus visits and job offers than candidates assigned to the control group, and that the effect on job offers was especially strong for women. It also finds, however, that these effects were more pronounced for candidates matched to influencers with relatively higher citation counts than for those matched to influencers with relatively more followers, as these two measures of prominence in the profession are not that highly correlated.

In this context, the authors make a welcome and useful contribution to the market design literature with a fascinating experiment designed to substitute for and even improve upon the informal information channels lost to the economics job market in the new postpandemic normal.

The question of scalability then becomes paramount. Considering the experiment’s positive findings, it is natural to assume that, if universally available, all job candidates would choose to participate and receive the treatment. The process would otherwise go on as stated with perhaps additional influencers being selected by the organizers to serve the larger pool of candidates. Two observations seem reasonable at this point: first, in such a setup, better information about matches could lead to more open positions being filled, which would be a better aggregate outcome; and second, the treatment might in practice benefit candidates from outside the very top departments the most. This is because candidates from the highest ranked departments, who are often perceived by recruiters as having a higher probability of eventually becoming a star, will typically receive more interviews, campus visits, and offers, but in the end can still only accept one offer. With an enlarged set of viable matches, this means that some candidates who may have been otherwise overlooked will find jobs. Of course, the job market may take longer to clear under this mechanism as candidates will have more options to consider before departments go to second or third rounds of offers.

Casual observations of the job market among economics departments and their chairs do suggest that a number of recruiters are unable to fill positions they have posted. The AEA does not currently collect information on just how many, but the very existence of the “AEA Job Market Scramble,” where recruiters and unmatched candidates can post their availabilities on an online message board each March, is indicative of the challenge (3). The design of a job signaling mechanism by the AEA and its implementation in December of each year (4), where job candidates can list two departments to which they would like to express interest in an interview, is another such intervention aimed at easing the congestion.

Another interesting result is that women appear to benefit most from the treatment, while this benefit does not extend to members of other groups traditionally underrepresented in economics. The authors point to existing evidence indicating that women on average tend to be less active promoters of their own research on social media than others and suggest that the additional visibility provided by the quote-tweets could be leading to more job offers. This potential channel, of course, could also be viable for any candidate with a tendency to self-promote less. To explain a special advantage for women, one could note the possibility of forces in the 2022–2023 job market where departments seeking to improve the gender balances of their faculties became aware of candidates through the mechanism who they may have otherwise overlooked. If this is the case, the next question to ask is why does the effect not carry over for members of other underrepresented groups? The answer, though no doubt a speculative one, may lie in the preexistence of other mechanisms and informal channels for promoting such candidates, rendering the marginal effects of the authors’ particular intervention not statistically significant.

Finally, while having the potential to increase the number of matches and raise their average quality, the effects of the authors’ intervention will be subject to some randomness based on the assignment of a given candidate’s influencer. For example, when any influencer posts a quote-tweet about a candidate who has been independently and objectively determined to have close research interests, that candidate’s post tends to receive more views and likes on X than those in the control group, and the extent of this visibility correlates with the size of the influencer’s following. Yet these effects do not seem to transfer downstream to job outcomes, where candidates receiving quote-tweets from highly cited influencers are the ones tending to see more offers. In a real sense, the adage “all publicity is good publicity,” often applied to economics research, may not be always true. The assignment of influencers to candidates, even if randomized, will matter for individual outcomes even though the aggregate effects of the intervention are positive. Given the potential individual benefits compared to nontreatment, however, job candidates would likely embrace the residual uncertainty and participate in the mechanism.

The intervention designed by Qiu et al. may hold even greater promise outside of the economics discipline. In the natural sciences, for example, recruiting for scarce academic postdoctoral positions among new PhDs at a similar career stage, which are markets typically saturated with candidates, often moves directly to a very limited allocation of campus visits based in no small part on letters and other communications from mentors, some of whom could be less than ideally matched with their students or less well known than would-be assigned influencers. These cases are ones in which an enhanced visibility of candidates, when coupled with independent information about the closeness of their work to what senior researchers and their groups might be seeking, could lead to the greater advancement of science more generally.
